# MicroRNA Profiling of Atrial Fibrillation in Canines: MiR-206 Modulates Intrinsic Cardiac Autonomic Nerve Remodeling by Regulating SOD1

**DOI:** 10.1371/journal.pone.0122674

**Published:** 2015-03-27

**Authors:** Yujiao Zhang, Shaohua Zheng, Yangyang Geng, Jiao Xue, Zhongsu Wang, Xinxing Xie, Jiangrong Wang, Shuyu Zhang, Yinglong Hou

**Affiliations:** 1 Department of Cardiology, Shandong Provincial Qianfoshan Hospital, Shandong University, No. 16766, Jingshi Road, Jinan, 250014, China; 2 School of Medicine, Shandong University, No. 44, Wenhua Xi Road, Jinan, 250012, China; 3 School of Radiation Medicine and Protection, Soochow University, No. 199, Renai Road, Suzhou, 215123, China; 4 Collaborative Innovation Center of Radiation Medicine of Jiangsu Higher Education Institutions, Soochow University, No. 199, Renai Road, Suzhou, 215123, China; Texas A& M University Health Science Center, UNITED STATES

## Abstract

**Background:**

A critical mechanism in atrial fibrillation (AF) is cardiac autonomic nerve remodeling (ANR). MicroRNAs (miRNAs) are small non-coding RNAs that regulate gene expression at the post-transcriptional level. Numerous miRNAs are involved in diseases of the nervous and cardiovascular systems.

**Objective:**

We aimed to assess the underlying role of miRNAs in regulating cardiac ANR in AF by right atrial tachypacing (A-TP) in canines.

**Methods and Results:**

Following 4-week A-TP, the superior left ganglionated plexuses (SLGPs), which are embedded in the fat pads of the left atrium, were subjected to miRNA expression profiling to screen preferentially expressed miRNAs. Sixteen miRNAs showed significantly differential expression between the control and A-TP groups, including miR-206, miR-203, miR-224 and miR-137. In particular, we focused on miR-206, which was elevated ~10-fold in A-TP dogs. Forced expression of miR-206 through lentiviral infection based on A-TP *in vivo* significantly shortened the atrial effective refractory period (AERP) (81 ± 7 *vs*. 98 ± 7 ms, *P* < 0.05). Immunohistochemical analysis showed that the regeneration of nerves increased more than 2-fold by miR-206 overexpression (*P* < 0.01). The expression of superoxide dismutase 1 (SOD1) was repressed by miR-206 overexpression by Western blot and luciferase assay, indicative of SOD1 as a direct target of miR-206. Overexpression of miR-206 increased reactive oxygen species (ROS) levels *in vitro* and *in vivo*, whereas miR-206 silencing attenuated irradiation- or A-TP-induced ROS. Knockdown of SOD1 effectively abolished ROS reduction caused by miR-206 silencing.

**Conclusions:**

Our results found the differential expression of miRNAs in response to ANR in AF and elucidated the important role of miR-206 by targeting SOD1. The study illustrated the novel molecular mechanism of ANR and indicated a potential therapeutic target for AF.

## Introduction

Atrial fibrillation (AF) is a highly prevalent disease associated with pronounced morbidity and mortality [1]. Accumulating evidence has demonstrated that cardiac autonomic nerve remodeling (ANR), characterized by nerve regeneration, as well as by uneven distribution of the sympathetic and vagus nerves, is involved in the pathogenesis of both acute and chronic AF [2, 3]. ANR and synergy with electricity and structural remodeling promotes one other to form a vicious cycle that induces AF [4, 5].

Cardiac nervous innervation involves both the extrinsic and intrinsic cardiac autonomic nervous systems (ANS). The former collectively includes the ganglia in the brain and along the spinal cord and their axons [6]. The latter consists of mainly of intrinsic cardiac ganglionated plexuses (GPs), which are usually embedded in the homologous fat pads (FPs) located on the epicardium of the atria of mammalian hearts, including the superior left GP (SLGP), the anterior right GP (ARGP), and the inferior right GP (IRGP) [7, 8]. The GPs are the distribution centers of nerves on the surface of the heart, and nerve regeneration in GPs is more intensive and obvious than in other places in the atrium [9]. Although atrial ANR in AF has been documented, ANR around the GPs and its biological mechanism on the molecular level have remained incompletely clarified.

MicroRNAs (miRNAs) are a class of endogenous non-coding small RNAs that maintain the balance of gene-regulating networks by binding to specific sites within the 3’UTR of target mRNAs to inhibit their expression. MiRNAs have been demonstrated to participate in many essential biological events and to be associated with the pathogenesis of various diseases, such as neurodegenerative diseases, tumors, muscle diseases, and cardiovascular diseases [10–13]. Additionally, it is reported that miRNAs may be involved in AF and the associated atrial remodeling process [14]. An abundance of evidence has indicated that the expression of miRNAs is distinctive in different parts of the central and peripheral nervous systems [15, 16]. Once a nerve is injured, the expression of miRNAs changes to adjust the corresponding target genes to promote nerve regeneration and repair [14].

Although the studies referenced above have provided promising evidence in support of the role of miRNAs in cardiac ANR, whether the expression of miRNAs is dysregulated during intrinsic cardiac ANS is unclear. In addition, the underlying molecular mechanisms of miRNAs on AF and the specific ANR in canine models remain unknown. We speculated that specific miRNAs were expressed in intrinsic cardiac ANS and affected cardiac ANR in AF via target downstream mRNAs. The present study was designed to clarify these questions.

## Materials and Methods

### 2.1 Canine Model of AF

This study conformed to Guidelines for the Care and Use of Laboratory Animals, published by the National Institutes of Health (NIH publication no. 85–23, revised 1996). The design and implementation of the study were approved by the Ethics Committee, Shandong University. Adult mongrel dogs (20 to 25 kg) of either sex were randomly divided into two groups: the control group (control, n = 6) and the atrial tachypacing group (A-TP, n = 6). The dogs were subjected to continuous A-TP at 400 bpm/min for 4 weeks in the A-TP group. The animals were intravenously anesthetized in a posterior lateral small saphenous vein with pentobarbital sodium (30 mg/kg) and were ventilated with room air by a positive pressure respirator. Permanent atrial pacemakers (AOO, made at Fudan University, Shanghai, China) were implanted in subcutaneous pockets and were attached to 4-electrode endocardial leads in the right atrial appendages via the jugular veins in the A-TP group. Surface electrocardiograms (ECGs) were recorded for AF by turning off the pacemaker every week. The tissue located < 0.5 cm around the LSFP was harvested after 4 weeks of A-TP.

### 2.2 MiRNA Expression Profiling

The total RNAs of the control and A-TP dogs were extracted from the left superior FP (LSFP) area using TRIzol reagent (Invitrogen, Carlsbad, CA, USA). Here, the fat tissues in the FPs were excluded as much as possible in the RNA collecting processes, to avoid mixing the adipose RNA. Equal quantities of RNAs from 3 dogs were mixed to generate one sample for each group to construct a small RNA library. The miRNA library was then sequenced with Illumina HiSeq sequencing systems (Illumina, San Diego, CA, USA). To identify miRNAs in canine FPs, genome-wide comparison of the sequences was conducted with the canine referenced genome sequences and all of the canine miRNAs in the miRBase database, version 19.0 (http://www.mirbase.org) (Genergy, Shanghai, China). The DESeq software used negative binomial distribution and a shrinkage estimator for the distribution’s variance to detect differential expression of miRNAs from high-throughput sequencing assays [17, 18].

### 2.3 Quantification of miRNA Expression

RNA was reverse transcribed to cDNA using a specific Reverse Transcription Kit for miRNA (D350, Takara, Tokyo, Japan), and quantitative real-time polymerase chain reaction (qRT-PCR) were conducted with Power SYBR Green (DRR081A, Takara, Tokyo, Japan). The forward primers for the miRNA amplification were designed based on the mature miRNA sequences (Table 1), and the reverse primers were provided by the manufacturer of the kit. MiRNA levels were quantified using ViiA7 (Applied Biosystems, CA, USA) with the 2^-ΔΔct^ relative quantification method [19].

**Table 1:** The Sequences of Primers of miRNAs.

Gene Name	Primer sequences
U6	Forward Primer 5’-TGCGTTGACGGTTTGACC-3’
miR-206	Forward Primer 5’-ACACTCCAGCTGGGTGGAATGTAAGGAAGT-3’
miR-203	Forward Primer 5’-ACACTCCAGCTGGGGTGAAATGTTTAGGAC-3’
miR-137	Forward Primer 5’-ACACTCCAGCTGGGTTATTGCTTAAGAAT-3’
miR-224	Forward Primer 5’-ACACTCCAGCTGGGCAAGTCACTAGTG-3’

### 2.4 Primary Myocardial Cell Isolation and Culture

Primary canine myocardial cells from < 0.5 cm around the LSFPs were isolated from the control canine myocardium following the procedures described previously [20]. Canine myocardial cells were maintained in Dulbecco’s modified Eagle’s medium (DMEM) supplemented with 10% fetal bovine serum and antibiotics (100 units/ml penicillin G and 100 units/ml streptomycin sulfate; Gibco, Grand Island, NY, USA).

### 2.5 Construction of Lentiviruses

The sequence of the mature miR-206 was 5’-UGGAAUGUAAGGAAGUGUGUGG-3’, and anti-miR-206 sequence was the exact antisense sequence of the mature miR-206. They were synthesized by TELEBIO Biotech (Shanghai, China). The recombinant fragment was separately inserted into an empty lentivirus, which was then co-transfected with three genomic plasmids (pRSV-Rev, pMDLg-pRRE, and pMD2G) in 293T cells. Lentivirus-mediated RNA interference (RNAi) targeting canine *SOD1* (*superoxide dismutase 1*, NCBI Reference Sequence: NM_001003035) was constructed by Genechem Biotech (Shanghai, China). The targeting region against *SOD1* is 5’-GACAATGAAGAAAGTACACAGACAG-3’. After 48 h of cultivation, the recombinant lentiviral vectors were prepared at a concentration of 1 × 10^9^ TU/ml for further experiments [21]. The lentiviruses containing scramble sequences and RNAi negative control (RNAi-NC) were separately constructed as miRNA control lentivirus (lenti-control) and RNAi-NC lentivirus (lenti-RNAi-NC).

### 2.6 Infection of Lentiviruses

*In vitro*, the cells were seeded (2 × 10^5^ cells/plate in 35 mm plate) in antibiotic-free medium 24 h prior to infection. Lentivirus of miR-206 overexpression (lenti-miR-206), miR-206 silencing (lenti-anti-miR-206) and SOD1 silencing (lenti-RNAi-SOD1) were used to infect cells for 48 h in different groups. Lentivirus was added with a multiplicity of infection (MOI) of 5 in cells. The lenti-control infected group and lenti-RNAi-NC infected group were used as controls.

For the *in vivo* studies, 30 mongrel dogs of either sex were randomly divided into 5 groups: 1) the lenti-control group: infection with control lentiviruses (n = 6), in which control lentiviruses were injected into LSFPs for 2 weeks without A-TP; 2) the lenti-miR-206 group: only infection with miR-206-overexpressed lentiviruses (n = 6), in which miR-206 lentiviruses were injected into LSFPs for 2 weeks without A-TP; 3) the lenti-anti-miR-206 group: only infection with miR-206 silencing lentiviruses (n = 6), in which anti-miR-206 lentiviruses were injected into LSFPs without A-TP; 4) the A-TP + lenti-miR-206 group: A-TP plus infection with miR-206-overexpressed lentiviruses (n = 6), in which miR-206 lentiviruses were injected into LSFPs after A-TP; 5) the A-TP + lenti-anti-miR-206 group: A-TP in combination with infection with anti-miR-206 lentiviruses (n = 6), in which anti-miR-206 lentiviruses were injected into LSFPs after A-TP. All of the dogs in each group underwent left thoracotomy at the fourth intercostal space, and the hearts were exposed. The left atrium was fixed, and 80 μl of lentiviruses (1 × 10^9^ TU/ml) were then directly injected into approximately 8 separate sites in the LSFP, which conveniently delivered the gene in a confluent, uniform area [21, 22]. Two weeks after infection, samples were collected for the quantification of miRNA and protein.

### 2.7 Detection of AERP and Inducibility of AF

The animals were intravenously anesthetized and exposed the hearts. The atrial effective refractory period (AERP) was measured in the posterior wall of left atrium with S1-S2 programmed electrical stimulation (PES): S2 at coupling intervals starting at 150 ms and progressively shortened in 10 ms decrements (S1:S2 = 8:1), 2 × diastolic threshold. The longest S1-S2 coupling interval that failed to result in a propagated atrial response was taken as the local AERP. AF was induced by PES with burst stimulation (cycle length 80 ms, lasting 2–3 minutes). Successful induction of AF was defined as a period of rapid irregular atrial rhythm lasting at least 30 seconds.

### 2.8 Immunohistochemical Analysis

Tissues were obtained from < 0.5 cm around the LSFPs in all of the animals. The nerve markers, including protein-gene-products 9.5 (PGP9.5), tyrosine hydroxylase (TH) and choline acetyltransferase (ChAT), were stained. The primary antibodies used were anti-PGP9.5 antibody (Abcam, Cambridge, UK; used at 1:30), anti-TH antibody (Pllabs, British Columbia, Canada; used at 1:500), and anti-ChAT antibody (Bioss, Beijing, China; used at 1:500). Each slide was examined under a microscope (Olympus, Tokyo, Japan) with a 20 × objective, and the 3 fields with the highest density of nerves were selected to calculate the mean nerve density. Nerve densities, which were expressed as the nerve area divided by the total area (μm^2^/mm^2^), were determined using a computer-assisted image analysis system (Image-Pro Plus 6.0, Media Cybernetics, Bethesda, MD USA).

### 2.9 Western Blot

The protein samples were extracted from the tissues located < 0.5 cm around LSFPs. Western blot was performed using primary antibodies against SOD1 (Abcam, Cambridge, UK) diluted to 1:250 and anti-GAPDH (Cell Signaling Technology, MA, USA) diluted to 1:1000. Western blot bands were quantified using Quantity One software (Bio-Rad, Hercules, CA, USA) by measuring the band intensity (area × OD) for each group. The final results are expressed as fold changes by normalizing the data to the control values [23].

### 2.10 Cell Transfection and Luciferase Assays

Luciferase vectors with wild-type (WT) SOD1 full-length 3’UTR (pGL3-SOD1-UTR-WT) or SOD1 full-length 3’UTR with a miR-206 binding site deletion (pGL3-SOD1-UTR-Del) were constructed by Shanghai Biobuy Biotech Co., Ltd. (Shanghai, China). The plasmids were confirmed by sequencing. Cells were transfected with the constructed vectors using Lipofectamine 2000 (Invitrogen, CA, USA). In each transfection, 50 ng of pRL-TK (Promega, Madison, WI, USA) was used to correct for the transfection efficiency. Luciferase activity was measured with the Dual-Luciferase Reporter Assay System (Promega). Promoter activity was expressed as the ratio of *Firefly* luciferase activity to *Renilla* luciferase activity.

### 2.11 ROS Generation Assay

The levels of reactive oxygen species (ROS) were determined using the ROS-sensitive dye 2’,7’-dichlorofluorescein diacetate (DCF-DA), which was converted by ROS into the highly fluorescent 2’,7’-DCF. Cells were incubated with DCF-DA (10 µM) for 30 min. The level of DCF fluorescence, which reflects the concentration of ROS, was measured. To induce ROS, the cells were irradiated 10 Gy of X-ray irradiation using a 6 MV X-ray linear accelerator (Clinac 2100EX; Varian Medical Systems, Inc., CA, USA) at a dose rate of 2 Gy/min; a 1.5-cm bolus was used as a compensator. After irradiation, the medium was immediately replaced with fresh DMEM medium. For the tissues, the level of DCF fluorescence was measured at 488 nm using a 96-well plate reader.

### 2.12 Statistical Analysis

The statistical analysis was performed using SPSS 17.0 software. Group data are expressed as means ± standard deviation (SD). The DESeq statistics were used to identify differentially expressed miRNAs between control and A-TP groups. The differences between two groups were analyzed using Student’s t-test, including quantification of miRNA and protein expression, luciferase assays, and part of fluorescence intensity. One-way analysis of variance was used when more than two groups were compared, including detection of AERP and immunohistochemical analysis. A result of *P* < 0.05 from a two-tailed test was interpreted to indicate a statistically significant difference.

## Results

### 3.1 MiRNAs Were Dysregulated in a Canine Model of AF

**Fig 1:**
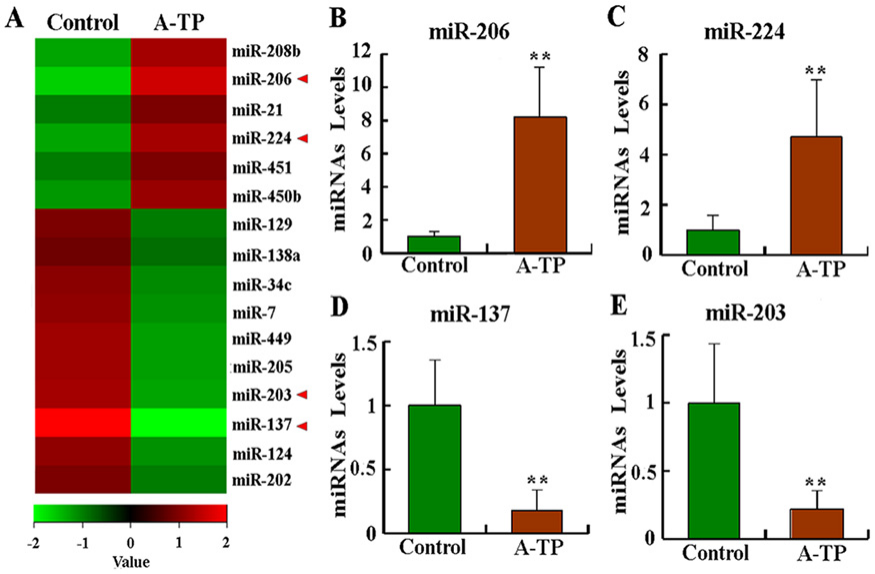
MiRNA expression profiling in the left superior FP (LSFP) subjected to atrial tachypacing (A-TP) in dogs. MiRNAs found to be significantly upregulated or downregulated in all tissues following A-TP compared with control samples were selected. (A) Heat map of miRNA profiles showing an increase or decrease in miRNA expression in the A-TP group. The coloring is standard; a red to green gradation in color represents higher to lower expression levels. The levels of miRNAs marked with red arrows were verified using quantitative assays. Detailed results of the levels of miRNAs in each layer of the hierarchical map are presented in Table 2. (B)- (E) Expression levels of selected miRNAs identified by expression profiling were independently determined using qRT-PCR. qRT-PCR data were normalized to U6. ** *P* < 0.01 compared with control samples.

The tissues from the control and A-TP dogs were analyzed for differentially expressed miRNAs by miRNA sequencing. All of the readings were aligned with the canine miRNAs in miRBase. A total of 241 miRNAs, out of 289 miRNAs measured, were found to be expressed in the two groups, of which 16 miRNAs were significant differentially between the control and A-TP groups (*P* < 0.05). The results revealed 6 miRNAs that were significantly upregulated and 10 miRNAs that were downregulated in the A-TP group, and they were subjected to hierarchical clustering (Fig 1A). The significantly dysregulated miRNAs in AF tissues included miR-206, miR-224, miR-137, miR-203, and so on (Fig 1A and Table 2). qRT-PCR analysis showed that miR-206, miR-224, miR-137 and miR-203 were all significantly dysregulated in A-TP tissues (*P* < 0.01, Fig 1B–1E). According to our sequencing data and bioinformatics analysis, miR-206 was significantly upregulated in AF tissues by ~10-fold over the lenti-control group. To observe the follow-up functional significance of miR-206, we were interested to investigate the consequences of miR-206 dysregulation. The raw sequencing data were submitted to the NCBI, and they are accessible at http://www.ncbi.nlm.nih.gov/sra/?term=SRX612537.

**Table 2:** Quantification of miRNA expression in A-TP group *vs*. Control group.

Gene Name	Absolute Fold change	Regulation	*P* value
cfa-miR-206	9.53	up	0.007
cfa-miR-208b	6.135	up	0.005
cfa-miR-21	3.86	up	0.016
cfa-miR-224	5.95	up	0.042
cfa-miR-451	3.83	up	0.017
cfa-miR-450b	5.00	up	0.041
cfa-miR-129	3.69	down	0.028
cfa-miR-138a	3.25	down	0.021
cfa-miR-34c	4.55	down	0.006
cfa-miR-7	4.86	down	0.028
cfa-miR-449	5.40	down	0.047
cfa-miR-205	5.68	down	0.002
cfa-miR-203	5.91	down	0.0002
cfa-miR-137	16.2	down	0.033
cfa-miR-124	4.73	down	0.043
cfa-miR-202	3.72	down	0.028

#Cfa-miRNA indicates the level of miRNA in canine model. DESeq statistical test was used to screen differentially expressed genes (*P* < 0.05)

### 3.2 Forced Expression of miR-206 Promoted Atrial ANR and AF

**Fig 2:**
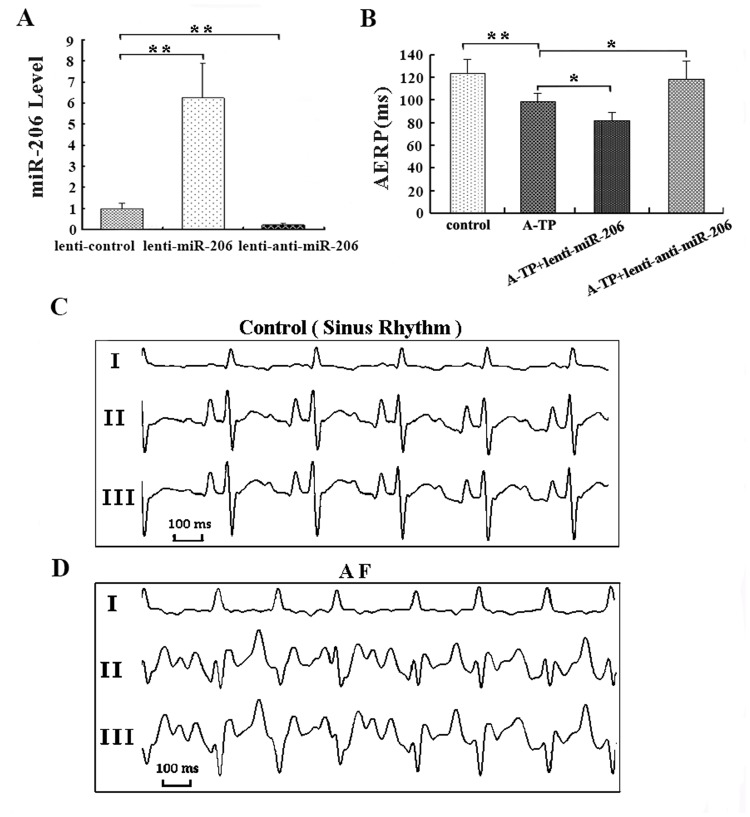
Characterization of atrial fibrillation (AF) and AERP in the canine model. AF was induced by A-TP for 4 weeks. During each week of A-TP, a surface electrocardiogram (ECG) was recorded to determine the presence of AF by turning off the pacemaker. The atrial effective refractory period (AERP) was measured with S1-S2 programmed electrical stimulation, with S2 at coupling intervals starting at 150 ms and progressively shortened by 10 ms decrements and with a 2 × diastolic threshold. The longest S1-S2 coupling interval that failed to result in a propagated atrial response was taken as the local AERP. (A) The level of miR-206 in the lenti-control, lenti-miR-206, and lenti-anti-miR-206 groups two weeks after infection. (B) The AERP in control, A-TP, A-TP plus lenti-miR-206 and A-TP plus lenti-anti-miR-206 groups obtained in posterior wall of left atrium. (C) ECG of the sinus rhythm obtained. (D) ECG of AF obtained. * *P* < 0.05 and ** *P* < 0.01.

A quantitative analysis of miR-206 levels was performed to test the efficacy of lentiviral infection after two weeks. Compared to the lenti-control group, the expression level of miR-206 was ~ 6-fold higher in the lenti-miR-206-infected dogs (*P* < 0.01), and it decreased to 20% in the lenti-anti-miR-206-infected group (*P* < 0.01, Fig 2A).

#### 3.2.1 miR-206 Influenced AERP and AF Inducibility

After 4 weeks of A-TP, the AERP was obviously shorter in the A-TP group than in the control group (98 ± 7 vs. 123 ± 12 ms, *P* < 0.01, Fig 2B), and it was even shorter in the A-TP plus lenti-miR-206 group (81 ± 7 vs. 98 ± 7 ms, *P* < 0.05, Fig 2B). In contrast, dogs treated with A-TP plus lenti-anti-miR-206 showed a significantly longer AERP than that in the A-TP group (118 ± 16 vs. 98 ± 7 ms, *P* < 0.05, Fig 2B).

Sinus rhythm and AF ECGs were detected from all the dogs (Fig 2C and 2D). After 4 weeks, AF was induced in 3 of 6 animals in the A-TP group, while AF did not occur in the control group. Furthermore, AF inducibility was increased to 5 of 6 in the A-TP plus lenti-miR-206 group and decreased to 1 of 6 in the A-TP plus lenti-anti-miR-206 group.

#### 3.2.2 miR-206 Modulated ANR

**Fig 3:**
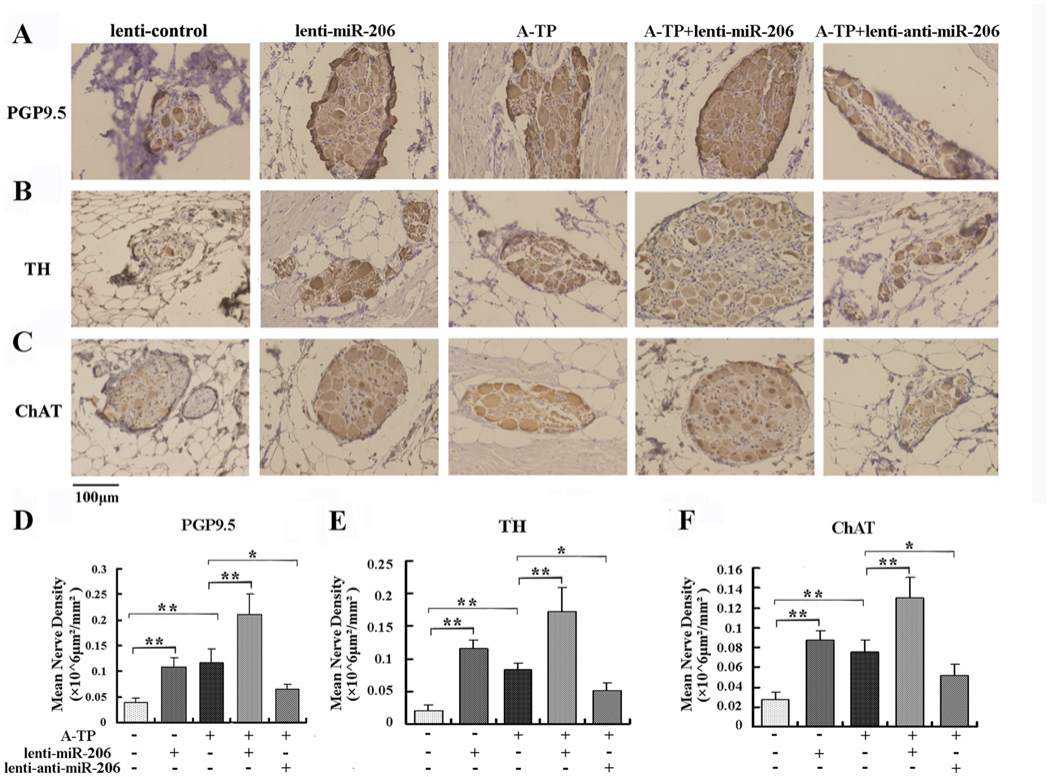
Measurement of nerve density by immunohistochemical analysis. (A) Immunohistochemical staining of PGP9.5-positive nerves in the SLGPs in the lenti-control, lenti-miR-206, A-TP, A-TP plus lenti-miR-206 and A-TP plus lenti-anti-miR-206 groups separately. (B) TH-positive nerves in five groups, as above. (C) ChAT-positive nerves in five groups, as above. (D) Histograms of mean PGP9.5-positive nerve density in the SLGPs. (E) Graphs of mean TH-positive nerve density. (F) Histograms of mean ChAT-positive nerve density. Magnification of the objective lens: 20 ×. Scale bar = 100 μm. * *P* < 0.05 and ** *P* < 0.01.

The nerve markers PGP9.5 (a marker of nerve sprouting), TH (a marker of sympathetic nerves), and ChAT (a marker of parasympathetic nerves) were used in response to ANR. Typical examples of sympathetic and parasympathetic nerve regeneration using the corresponding markers are shown in Fig 3. Immunohistochemical staining showed that the densities of PGP9.5-, TH- and ChAT-positive nerves in the SLGPs were all significantly increased in A-TP dogs, compared to the lenti-control group (*P* < 0.01, Fig 3). When lenti-miR-206 was infected, the mean nerve densities of PGP9.5-, TH- and ChAT-positive nerves were all increased more than 2-fold, compared to the lenti-control dogs (*P* < 0.01, Fig 3). In the A-TP plus lenti-miR-206-infected dogs, the PGP9.5-, TH- and ChAT-positive nerves were significantly increased, compared to the A-TP group (*P* < 0.01, Fig 3). However, the opposite results were obtained in the A-TP in combination with lenti-anti-miR-206 group (*P* < 0.05, Fig 3). In addition, there were no obvious differences between sympathetic and parasympathetic nerves. Taken together, these results indicated A-TP and overexpression of miR-206 both induced the ANR in canine LSFPs.

#### 3.2.3 SOD1 Was a Direct Target for miR-206

**Fig 4:**
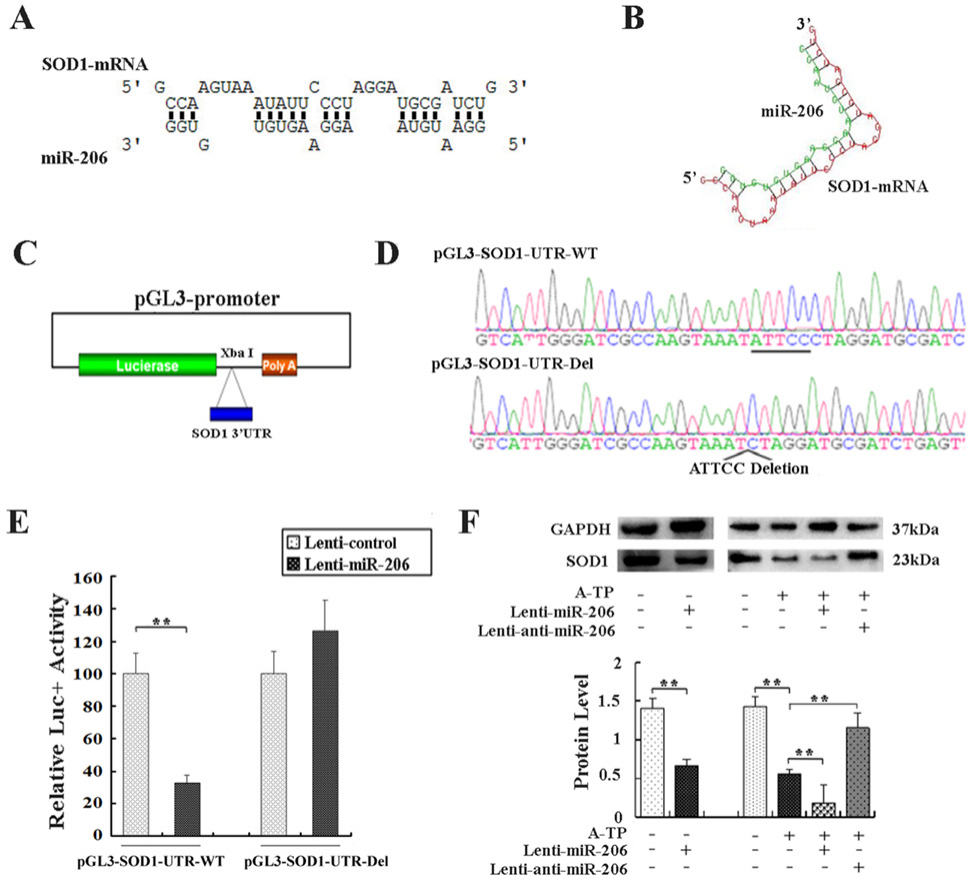
SOD1 was a direct target of miR-206. (A) Putative miR-206-binding sequences in the 3’UTR of SOD1 mRNA, identified by RNAhybrid 2.2. (B) The predicted miR-206-SOD1 binding structure, identified by RNAhybrid 2.2. (C) Schematic diagram of constructed vectors. The 3’UTR region of SOD1 was cloned downstream of the luciferase reporter gene (pGL3-promoter vector). (D) The sequence of the luciferase vector in pGL3-SOD1-UTR-WT and pGL3-SOD1-UTR-Del. (E) Primary canine myocardial cells were transfected with *Firefly* luciferase expression vectors with pGL3-SOD1-UTR. Luciferase activity was assayed 24 h after transfection. The *Firefly* luciferase activity of each sample was normalized to *Renilla* luciferase activity. The normalized luciferase activity of the cells transfected with the control lentivirus was set as 100% relative luciferase activity. The column graphs show the means of at least three independent experiments performed in duplicate. (F) Tissues of the LSFPs were collected in each group. SOD1, and internal standard GAPDH protein levels were detected as above. Western blot bands were normalized to GAPDH. **P < 0.01.

Bioinformatics algorithms (TargetScan and RNAhybrid) were used to explore the possible miRNA targets. One binding site for miR-206 was predicted in the proximal 3’UTR of SOD1 (Fig 4A and 4B), indicating that SOD1 might be a mimR-206 target gene. Two luciferase vectors were constructed: pGL3-SOD1-UTR-WT and pGL3-SOD1-UTR-Del (Fig 4C and 4D). As shown in Fig 4E, overexpression of miR-206 by lenti-miR-206 caused a substantial reduction of luciferase activity in pGL3-SOD1-UTR-WT-transfected cells (*P* < 0.01), suggesting that miR-206 could negatively regulate SOD1. This decrease was prevented by the deletion of the predicted miR-206 binding site in the SOD1 3’UTR, indicating that the SOD1 proximal 3’UTR contained one miR-206 binding site (Fig 4E). Therefore, these data confirmed that SOD1 was the target gene of miR-206 in canines.

*In vivo* results from Western blot analysis showed that forced expression of miR-206 repressed the expression of SOD1 to approximately 48% of that in the lenti-control group (*P* < 0.01, Fig 4F). The protein level of SOD1 in A-TP dogs was downregulated to approximately 39% of that in the lenti-control group (*P* < 0.01), and the level of SOD1 in the A-TP plus lenti-miR-206 group decreased to approximately 34% of that in the A-TP tissues (*P* < 0.01). However, the downregulation was efficiently abolished by lenti-anti-miR-206 infection, which increased to ~2.1-fold of that in the A-TP tissues (*P* < 0.01).

#### 3.2.4 miR-206 Regulated ROS via SOD1

**Fig 5:**
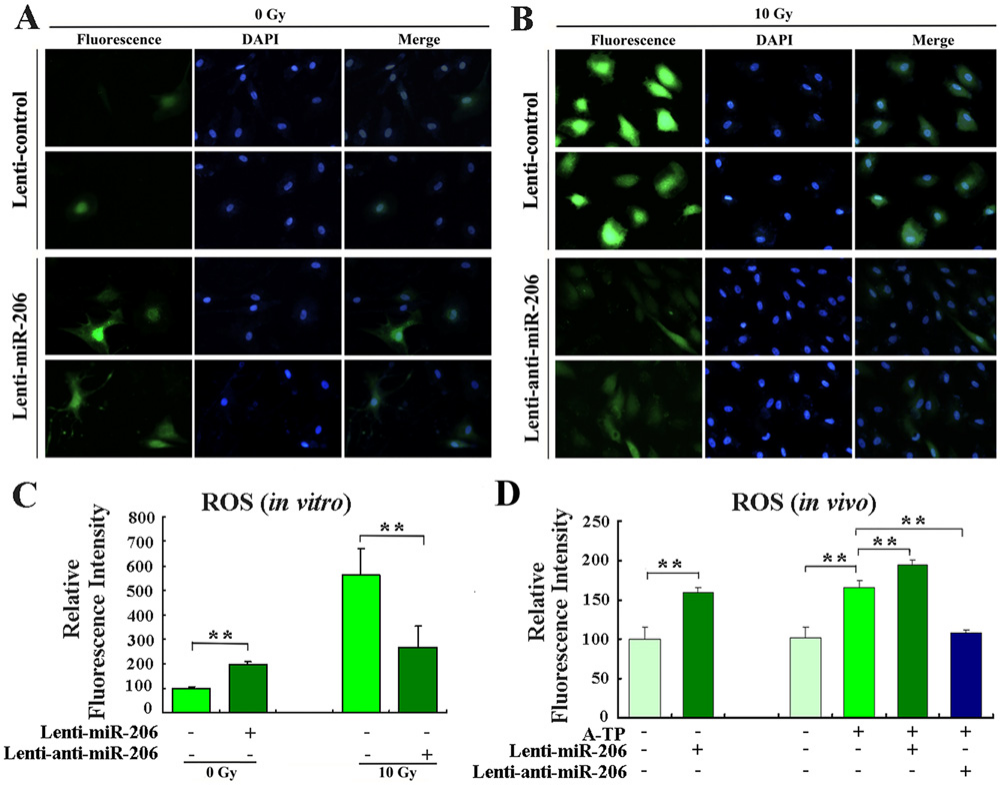
miR-206 regulated ROS levels *in vitro* and *in vivo*. (A) Determination of ROS baseline levels of primary canine myocardial cells infected or not with lenti-miR-206. (B) To induce ROS, cells infected or not with lenti-anti-miR-206 exposed to 10 Gy of X-ray irradiation. Forty-eight hours after infection, the ROS levels were determined using the ROS-sensitive dye 2’,7’-dichlorofluorescein diacetate (DCF-DA). Fluorescent signals, reflecting the concentration of ROS, were measured by a fluorescence microscope under the same conditions. (C) Relative ROS levels in indicated groups of cells, as calculated by Image J analysis software (MD, USA). The normalized fluorescent signals of the cells infected with the control lentivirus were set as 100. (D) ROS levels were determined using DCF-DA in tissues. Dogs were injected with control lentivirus or the miR-206-overexpressing lentivirus. Two weeks after infection, the ROS levels in fresh tissues were measured. The level of DCF fluorescence was measured at 488 nm using a 96-well plate reader. **P < 0.01.

*In vitro*, canine myocardial cells were infected with lenti-control, lenti-miR-206 or lenti-anti-miR-206 to determine whether miR-206 affected ROS levels. At 48 h after lentivirus infection, the ROS levels in the cells were measured. Infection of lenti-miR-206 increased the baseline level of cellular ROS by ~ 2-fold (*P* < 0.01, Fig 5A and 5C). To induce cellular ROS, cells were exposed to 10 Gy of X-ray irradiation. The infection of lenti-anti-miR-206 significantly reduced the level of radiation-induced ROS to approximately 50% of that in the lenti-control group (*P* < 0.01, Fig 5B and 5C).

*In vivo*, similar results were obtained. The ROS level of lenti-miR-206-infected dogs was ~ 1.6-fold of the lenti-control dogs (*P* < 0.01, Fig 5D). The ROS level was obviously higher in the A-TP group than that in the lenti-control dogs (*P* < 0.01). Additionally, the level of ROS was significantly increased in the A-TP in combination with lenti-miR-206 group compared to the A-TP dogs (*P* < 0.01), and it was efficiently reversed by infection with lenti-anti-miR-206 (*P* < 0.01). Taken together, these results indicated that miR-206 upregulated the level of ROS by suppressing SOD1 expression.

**Fig 6:**
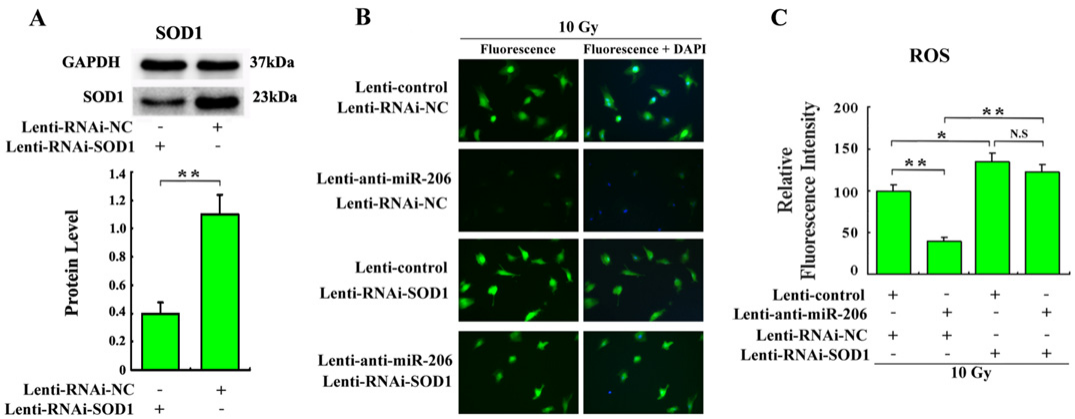
miR-206 regulated ROS via SOD1 in canine primary myocardial cells. (A) The canine myocardial cells were infected with lenti-RNAi-NC or lenti-RNAi-SOD1. 48 h after infection, cells were collected and SOD1 and internal standard GAPDH protein levels were detected by Western blot. **P < 0.01. (B) Determination of ROS levels of canine primary myocardial cells infected with indicated lentiviruses. Cells were infected with lenti-control, lenti-RNAi-NC, lenti-anti-miR-206 or lenti-RNAi-SOD1. To induce ROS, cells were exposed to 10 Gy X-ray irradiation. 48 h after infection, the ROS levels were determined. Representative images in each group by a fluorescence microscope were under the same condition. (C) Relative ROS levels in indicated groups were calculated. * *P* < 0.05, ** *P* < 0.01 and N.S: not statistically significant.

Our data suggested that overexpression of miR-206 induced an ROS increase and that SOD1 was a direct target of miR-206. Thus, we were interested to determine whether miR-206 regulated ROS by mediating SOD1. Lentivirus targeting-SOD1 was constructed and could silence the expression of SOD1 to approximately 36% in myocardial cells (*P* < 0.01, Fig 6A). SOD1 knockdown by lenti-RNAi-SOD1 infection in myocardial cells resulted in upregulation of ROS levels to approximately 1.4-fold (*P* < 0.05, Fig 6B and 6C). However, the ROS levels were showed no significant difference between the group of SOD1 knockdown and the group of co-infection of lenti-RNAi-SOD1 and lenti-anti-miR-206 in myocardial cells (*P* > 0.05, Fig 6B and 6C). In addition, lenti-anti-miR-206-mediated ROS reduction was reversed by SOD1 silencing, which verified that miR-206 regulated ROS levels by target-SOD1.

## Discussion

In the present study, we found that multiple miRNAs were related to AF induced by A-TP in dogs. Of the identified miRNAs, we focused on miR-206, which was significantly upregulated in A-TP dogs. It was shown that the AERP was significantly shortened, and the inducibility of AF had an obvious tendency to increase by A-TP and miR-206 upregulation. The mean nerve density in the LSFPs was increased by A-TP, and overexpression of miR-206 enhanced nervous hyperinnervation, while anti-miR-206 exerted exactly the opposite effect. Forced expression of miR-206 could boost ROS levels by downregulation of SOD1 *in vitro* and *in vivo*. Taken together, these results demonstrated that the overexpression of miR-206 promoted ANR by targeting SOD1 and production of ROS.

Various miRNAs have been reported to be closely related to AF [24–26]. In previous studies, arrhythmogenic miRNAs, including miR-1, miR-21, miR-133, miR-590, miR-328, etc., were found to participate in different mechanisms of AF [14, 21, 24–26]. Among these miRNAs, the regulation of miR-1, miR-133 and miR-328 contributes to electrical remodeling in AF, whereas miR-21 and miR-590 contributed to structural remodeling in AF. To search for miRNAs that might be involved in the progression of ANR induced by A-TP in dogs, differentially expressed miRNAs were obtained by miRNA sequencing. The results of the miRNA expression profile suggested that plenty of miRNAs might be involved in AF. Our result was primarily consistent with the prior studies in the development of heart disease, including miR-137, miR-21, miR-208b, miR-451, miR-124, and so on [24, 27–30]. In particular, the expression of miR-206 was significantly upregulated in the A-TP model. miR-206 has exhibited its regulation of fibroblast growth factor, Hmgb3 and HIF-1α/Fhl-1 pathways in amyotrophic lateral sclerosis, muscle regeneration and pulmonary hypertension, respectively [31–33]. miR-206 has also been found to accelerate nerve regeneration [34]. Therefore, miR-206 was possible to be involved in the occurrence and development of AF. Our results extend the roles of miR-206 on ANR in AF.

In the early period of AF, characteristic changes in the AERP and a loss of rate adaptation occur. Recently, the abnormal expression of miRNAs has attracted the interest regarding the molecular mechanism of underlying cardiac electrophysiological properties, including these AERP changes and vulnerability to AF [21]. The AERP was significantly shortened by A-TP or overexpression of miR-206 in the present work. Although the inducibility of AF by miR-206 was not significantly different, it suggested that the role of overexpression of miR-206 had a tendency toward increasing AF inducibility.

Recent evidence has shown that dogs with AF undergo heterogeneous changes in atrial sympathetic innervation [2, 35]. Similarly, significant parasympathetic nerve remodeling has also been demonstrated in the right and left atria in canine models of AF induced by 48 h of right atrial pacing [3]. The present study showed that AF induced by sustained A-TP was accompanied by sympathetic and vagus hyperinnervation in the SLGPs by nerve indicators PGP9.5, TH and ChAT. Nerve remodeling in GPs might be a more important cause of the initiation and maintenance of AF than that in other part of the atrium. Changes of the level of miR-206, which is preferentially expressed in muscles and nerves, were accompanied by nerve proliferation, including of the central and peripheral nerves. In the period after acute spinal cord injury and sciatic nerve transection injury, miR-206 was significantly upregulated [15, 36]. In our study, forced overexpression of miR-206 increased the densities of the sympathetic and parasympathetic nerves, indicating that miR-206 promoted nerve remodeling. It was speculated that upregulation of miR-206 in all of the FPs tissues might boost ANR in AF by directly regulating its target SOD1 and other pathways.

Previous report suggested that, SOD1 was a target of miR-206 and the mutant of SOD1 induced the nerve remodeling [37]. Notably, the context of this study was the observation that SOD1 was a downstream effector of miR-206. SOD1 contributes to approximately 70–80% of cellular SOD activity, and it plays a pivotal role in the modulation of sympathetic nerve activity [38]. SOD1 overexpression was shown to attenuate excessive nervous activation in the nervous system and in myocardial remodeling in a previous report [39]. We found that SOD1, as a direct target of miR-206, was attenuated by overexpression of miR-206 or intervention of A-TP. Moreover, the effect of miR-206 in ROS regulation was mediated by SOD1. Numerous studies have demonstrated that SOD1 is crucial in the elimination of cellular ROS including H_2_O_2_, O_2_- and^.^ OH from metabolites [40]. Experimental and clinical studies have suggested that ROS play a role not only as triggers of AF but also as factors maintaining cardiac arrhythmia once AF has developed [41–43]. Several pathways seem to be implicated in ROS overproduction, ultimately leading to enhanced vulnerability to AF. ROS have been found to increase neuronal differentiation in PC12 cells in neurogenesis [44, 45]. Furthermore, Numb-interacting protein 1, a novel intrinsic regulator of neuronal cell fate, guided neuronal development through ROS generation [46]. These evidences indicated that ROS induced neurogenesis and promoted neuronal cell development. In the present study, the dysregulation of miR-206 in local tissues might have resulted in the changes in surrounding ROS levels by intercellular transport, which could distribute ROS to adjacent nervous cells and affect these cells. Consequently, the overexpression of miR-206 might promote ROS generation by reducing the expression of the antioxidant SOD1 protein, potentially inducing nerve remodeling in SLGPs.

Our experiments also had limitation. A total of 16 differentially expressed miRNAs were identified by miRNA expression profiling in the present study, but we only focused on the function of miR-206. Although the results support a role of miR-206 in these biological processes, the possibility of other functional miRNAs could not be excluded.

In conclusion, our results showed that multiple miRNAs were differentially expressed in response to ANR in AF. miR-206 overexpression induced cardiac ANR and the changes of electrophysiological properties to facilitate AF. The underlying mechanism was that miR-206 increased the production of ROS by targeting SOD1. Thus, this study provided a detailed characterization of the effects of miRNAs on ANR, and it suggested a potential therapeutic target for AF.
